# Hepcidin in morbidly obese women with non-alcoholic fatty liver disease

**DOI:** 10.1371/journal.pone.0187065

**Published:** 2017-10-24

**Authors:** Teresa Auguet, Gemma Aragonès, Alba Berlanga, Salomé Martínez, Fàtima Sabench, Jessica Binetti, Carmen Aguilar, José Antonio Porras, Alicia Molina, Daniel Del Castillo, Cristóbal Richart

**Affiliations:** 1 Grup de Recerca GEMMAIR (AGAUR)- Medicina Aplicada, Departament de Medicina i Cirurgia, Universitat Rovira i Virgili (URV), Institut d’Investigació Sanitària Pere Virgili (IISPV), Tarragona, Spain; 2 Servei de Medicina Interna, Hospital Universitari Joan XXIII, Tarragona, Spain; 3 Servei d’ Anatomia Patològica, Hospital Universitari Joan XXIII, Tarragona, Spain; 4 Servei de Cirurgia, Hospital Universitari Sant Joan, Reus, Departament de Medicina i Cirurgia, Universitat Rovira i Virgili (URV), IISPV, Reus, Spain; Medizinische Fakultat der RWTH Aachen, GERMANY

## Abstract

**Background:**

Non-alcoholic fatty liver disease (NAFLD) is the most common cause of chronic liver disease in Western countries. Both iron and lipid metabolism seem to be involved in its pathogenesis. We aimed to assess the relationship between levels of hepcidin, the master iron-regulatory protein, in plasma and the presence of NAFLD in morbidly obese (MO) patients, and to investigate the association between the hepatic expression of the main iron and lipid metabolism -related genes.

**Materials and methods:**

Enzyme-linked immunosorbent assay was used to measure plasma hepcidin levels in 49 normal-weight control women, 23 MO women with normal liver (NL) histology and 46 MO women with NAFLD. The mRNA expression of hepcidin, the main iron metabolism-related genes, and the main lipid-metabolism genes was quantified by qRT-PCR in liver biopsies from members of the MO group undergoing bariatric surgery.

**Results:**

Circulating hepcidin levels were significantly greater in MO than in normal-weight control women. However, there were no significant differences between MO women with NL and those with NAFLD. PCR analysis showed increased expression of hepcidin, FPN1, TfR1 and TfR2 in the liver of MO NAFLD women compared to those with NL. Moreover, a positive association of hepatic hepcidin mRNA expression and the iron metabolism-related genes was found with some key genes involved in the lipid metabolism.

**Conclusion:**

These findings suggest that circulating hepcidin levels are associated with obesity but not with the presence of NAFLD. However, the hepatic expression of hepcidin and the iron metabolism-related genes seem to play a role in regulating lipid metabolism pathways in liver, which has implications for NAFLD pathogenesis.

## Introduction

Non-alcoholic fatty liver disease (NAFLD) is a wide spectrum liver disease, ranging from simple steatosis (SS) to non-alcoholic steatohepatitis (NASH). While SS is considered to be a relatively benign, non-progressive clinical entity, NASH can progress to cirrhosis and, in a small percentage of patients, to hepatocellular carcinoma [1]. The current estimate of the global prevalence of NAFLD is 25.24% and is quite high in patients with components of metabolic syndrome. For instance, over 90% of severely obese patients undergoing bariatric surgery have NAFLD [2].

The accumulation of fat in the liver, which occurs in the absence of significant alcohol consumption, is a key feature of NAFLD. It has been reported that the mechanisms leading to excessive hepatic lipid accumulation arise from an imbalance between lipid acquisition and removal, a process in which insulin resistance and lipid metabolism deregulation play a key role [3,4]. Although lipid accumulation alone may be benign, more aggressive forms of NAFLD can develop as a result of other mechanisms. In this regard, the most generally accepted hypothesis at present to explain the progression from SS to NASH is the “multiple hit” hypothesis, which considers multiple insults acting together, including hormones secreted from the adipose tissue, oxidative stress, mitochondrial dysfunction, gut microbiota, genetic and epigenetic factors, and iron overload [5–8]. In the pathogenesis of NAFLD, iron is likely to induce oxidative stress, mitochondrial dysfunction and inflammation in the liver [9,10]. Serum iron indices and hepatic iron content are frequently shown in patients with NAFLD/NASH [11,12]. In fact, clinical studies with NAFLD patients have shown a clear association between hepatic iron deposition and disease severity [9,13–15]. Moreover, liver damage has been shown to improve after iron reduction therapy in patients with NAFLD [16,17].

The liver is the major site of iron deposition and has a central role in iron homeostasis because it regulates hepcidin production, the master iron-regulatory protein which is primarily synthesized in hepatocytes and is found in human serum and urine [18]. Hepcidin controls iron release from spleen macrophages and hepatocytes, and also dietary iron absorption from enterocytes by degrading ferroportin (FPN), the only known iron exporter in mammals [19,20]. Its hepatic expression is regulated by a combination of factors such as iron concentrations, inflammation, erythropoiesis, and sex hormone. Important up-stream regulators include hemojuvelin (HJV), hemochromatosis protein (HFE), and transferrin receptor 1 and 2 (TfR1/TfR2) [21]. Hepcidin expression is also enhanced by increased iron stores via bone-morphogenic protein receptor (BMPR) and Sma and Mad related proteins (SMAD) (HJV/BMPR/SMAD pathway) and also via induction of Janus kinase/Signal transducer and activator of transcription (JAK2/STAT3) pathway when inflammation exists. Commonly, these conditions are present in NAFLD [22,23]. On the other hand, hepcidin is inhibited by anemia and hypoxia [24].

The role of hepcidin as a non-invasive biomarker for NASH or NAFLD has generated considerable interest since some studies have shown variable increase in its serum levels in patients with NAFLD and variable correlations with hepatic inflammation and histological severity [25–27]. However, the role it plays in the pathogenesis of NAFLD still remains controversial [28–31]. Some authors have suggested that more severe forms of NAFLD are associated with insufficient hepcidin production [28,32]. Moreover, in a recent study, Handa et al., observed elevated hepatic hepcidin expression in patients with NASH and in NAFLD patients who had hepatic iron deposition, while proinflammatory cytokines displayed elevated expression only in patients with NASH, suggesting a regulatory role for hepcidin in NAFL to NASH transition and in mitigating inflammatory responses [23].

Finally, some studies have shown that lipid metabolism might be involved in hepcidin synthesis [29].

With the hypothesis that hepcidin have a regulatory role in the progression of NAFLD, the first aim of the present study was to evaluate the relationship between plasma hepcidin levels and the presence of NAFLD in a cohort of morbidly obese women with NAFLD. In addition, as lipid metabolism seems to be involved in the pathogenesis of NAFLD and may be related to hepcidin synthesis, our second aim was to investigate the association between the hepatic expression of the main genes involved in iron metabolism and the expression of lipid metabolism-related genes.

## Material and methods

### General protocol

This study was approved by the institutional review board (*Comitè d’Ètica d’Investigació Clínica*, *Hospital Universitari Joan XXIII de Tarragona*, 23c/2015), and all participants gave written informed consent for participation in medical research. We included 69 morbidly obese (MO) women and 49 normal-weight control women, with a body mass index (BMI) greater than 40 kg/m^2^ and less than 25 kg/m^2^, respectively.

Liver biopsies were only obtained from the MO group during planned bariatric surgery and were always performed for diagnostic indications. The weight of all subjects was stable for at least three months prior to surgery. The diagnosis of NAFLD was made on the basis of the following criteria: liver pathology, and an intake of less than 10 gr. of ethanol/day. The exclusion criteria were: (1) concurrent use of medications known to produce hepatic steatosis or hormone replacement therapy or herbal products (2) history of hepatotoxic drugs, (3) patients using lipid-lowering medications including PPARα or γ- agonists, (4) menopausal and post-menopausal women and subjects receiving contraceptive treatment, (5) patients who had viral hepatitis or autoimmune hepatitis, hemochromatosis, Wilson’s disease, alpha-1 antitrypsin deficiency, biliary disease, anemia, impaired renal function, current evidence of acute or chronic inflammatory diseases potentially capable of causing hyperferritinemia and end-stage malignant diseases.

### Histological evaluation

All liver samples were scored by experienced hepatopathologists using the methods described elsewhere [33,34]. Simple steatosis (SS) was graded as follows: Grade 1 or mild SS: more than 5% and less than 33% of hepatocytes affected; Grade 2 or moderate SS: 33% to 66% of hepatocytes affected; or Grade 3 or severe SS: more than 66% of hepatocytes affected. Moreover, the minimum criteria for the steatohepatitis diagnosis included the presence of either ballooning cells and lobular inflammation or perisinusoidal /pericellular fibrosis in zone 3 of the hepatic acinus. According to their liver pathology and BMI, MO patients were sub-classified into the following groups: (1) MO women with normal liver (NL) histology (n = 23); (2) MO women with simple steatosis (SS) (micro/macrovesicular steatosis without inflammation or fibrosis, n = 22); (3) MO women with non-alcoholic steatohepatitis (NASH) (Brunt grade 1–3, n = 24).

### Laboratory evaluation

All patients were evaluated by a complete anthropometrical, biochemical, and physical assessment. Body height and weight were measured with the subjects standing in light clothes and shoeless. BMI was calculated as body weight divided by height squared (Kg/m^2^). Patient’s waist circumference (WC) was measured with a soft tape midway between the lower rib and the iliac crest.

Laboratory analysis included circulating levels of glucose, insulin, glycosylated haemoglobin (HbA1c), high-density lipoprotein cholesterol (HDL-C), low-density lipoprotein cholesterol (LDL-C), triglycerides (TG), transaminases and circulating iron status (including ferritin, transferrin, transferrin saturation and iron levels), which were measured using a conventional automated analyzer after overnight fasting. Insulin resistance was estimated using the homeostatic model assessment 2-insulin resistance (HOMA2-IR) [35]. We also analyzed plasma hepcidin concentrations, measured by a commercially available enzyme-linked immunosorbent assay (ELISA) kit (DRG Hepcidin 25 (bioactive) HS ELISA) (Catalog No: EIA-5782, DRG Diagnostics, Marburg, Germany), according to the manufacturer’s instructions. The detection limit was 0.153 ng/mL. The intra- and inter-assay coefficients of variation were between 5 and 15%.

### RNA Isolation and Real-time PCR

Liver biopsy sample were preserved in RNA later (Sigma, San Louis, MO, USA) for 24 h at 4°C and then stored at -80°C until use. Total RNA was isolated from liver by using an RNeasy mini kit (Qiagen, Barcelona, Spain) in accordance with the manufacturer’s protocol. First-strand cDNA was obtained from total RNA using the High Capacity RNA-to-cDNA Kit (Applied Biosystems, Madrid, Spain). Real-time quantitative PCR was carried out with TaqMan Assays predesigned by Applied Biosystems (Foster City, CA, USA) for the detection of hepcidin (HAMP, Hs00221783_m1), ferroportin 1 (FPN1, Hs00205888_m1), hemojuvelin (HJV, Hs00377108_m1), transferrin receptor 1 (TfR1, Hs00951083_m1), transferrin receptor 2 (TfR2, Hs01056398_m1), LXRa (Hs00173195_m1), SREBP1c (Hs01088691_m1), ACC1 (Hs00167385_m1), FAS (Hs00188012_m1), PPARa (Hs00947538_m1), CPT1a (Hs00912671_m1), CROT (Hs00221733_m1), SREBP2 (Hs01081784_m1), ABCA1 (Hs01059118_m1), ABCG1 (Hs00245154_m1), and 18S ribosomal RNA (4333760T) that was used as a housekeeping gene. All reactions were carried out in duplicate in 96-well plates using the 7900HT Fast Real-Time PCRsystems (Applied Biosystems).

### Statistical analysis

Statistical analyses were performed using SPSS 23.0 statistical package for Windows (IBM Corporation, New York, NY, USA). All values were tested for normality using the Kolmogorov—Smirnov test. Data are presented as the median (25th percentile -75th percentile). Differences between the study groups were calculated using non-parametric test: Mann—Whitney U-test and Kruskal-Wallis analysis. The strength of association between the study variables was analyzed by the Spearman’s tests. *p*-Values <0.05 were considered to show statistically significant results.

## Results

### Baseline characteristics of subjects

The main characteristics of the study cohort, including anthropometric and biochemical (liver enzymes, and glucose, lipid and iron metabolism) parameters are shown in Table 1. Our cohort of 118 women was classified according to the body mass index (BMI) and hepatic histology into normal-weight (n = 49), morbidly obese with normal liver histology (NL, n = 23), and morbidly obese with NAFLD (n = 46). In terms of age and anthropometric measurements (weight, BMI and waist circumference [WC]), there were no significant differences between NL and NAFLD in the morbidly obese group. Biochemical analyses indicate that insulin and triglycerides were also significantly lower in normal-weight women than in NL and NAFLD MO women. Although levels of glucose, homeostatic model assessment 2-insulin resistance (HOMA2-IR) and glycosylated haemoglobin (HbA1c) were not significantly different between normal-weight women and MO women with NL histology, its circulating levels were significantly lower in normal-weight women compared to MO women with NAFLD. When we compared liver histologies in the morbidly obese group, we observed that insulin, triglycerides, glucose, HOMA2-IR, and HbA1c were significantly greater in NAFLD than in NL subjects. Moreover, circulating levels of high-density lipoprotein cholesterol (HDL-C) were significantly higher in normal-weight women than in NL and NAFLD MO subgroups, and significantly lower in MO women with NAFLD than in those with NL histology. As far as transaminases are concerned, Table 1 showed that levels of aspartate aminotransferase (AST), alanine aminotransferase (ALT) and alkaline phosphatase (ALP) were significantly higher in MO women with NAFLD than in normal-weight and MO women with NL histology. However, levels of gamma-glutamyltransferase (GGT) were not significantly different between NL and NAFLD in the morbidly obese group, although their circulating levels were significantly greater in MO women with NAFLD than in normal-weight control women. Finally, with regard to iron metabolism parameters, we observed significantly higher levels of iron and transferrin in normal-weight women than in MO women with NAFLD. However, hepcidin levels in both MO women with NAFLD and NL histology were significantly higher than in normal-weight subjects. We also observed that ferritin levels were significantly greater in MO NAFLD women than in MO with NL histology and normal-weight women. Regarding inflammation, CRP levels in both MO women with NAFLD and NL were significantly higher than in normal-weight subjects.

**Table 1:** Clinical characteristics of the study cohort classified according to the BMI and histopathological characteristics.

Variables	Normal-Weight	Morbidly Obese *(n = 69)*	
	*(n = 49)*	NL *(n = 23)*	NAFLD *(n = 46)*
**Age (years)**	43.9 (34.6–52.2)	45.1 (40.3–58.8)	48.3 (44.6–54.8)
**Weight (Kg)**	59.0 (52.2–64.0)	120.0 (112.0–131.0)*	120.0 (112.5–129.3)*
**BMI (Kg/m2)**	23.1 (21.6–24.2)	47.5 (42.0–52.7)*	46.8 (44.2–51.4)*
**WC (cm)**	74.0 (69.5–79.5)	134.0 (121.8–149.3)*	133.0 (125.0–139.0)*
**Glucose (mg/dL)**	82.5 (69.0–91.0)	84.0 (76.8–96.8)	116.0 (102.0–152.0)*^#^
**Insulin (mUI/L)**	6.0 (4.2–9.9)	9.7 (7.9–13.1)*	17.3 (9.3–25.4)*^#^
**HOMA2-IR**	0.7 (0.5–1.3)	1.2 (1.0–1.6)	2.6 (1.4–3.5)*^#^
**Total cholesterol (mg/dL)**	180.0 (169.9–202.9)	163.2 (143.8–204.0)	178.9 (152.3–206.9)
**HDL-C (mg/dL)**	60.6 (50.8–65.3)	43.9 (35.8–54.3)*	38.0 (34.9–43.8)*^#^
**LDL-C (mg/dL)**	110.3 (93.9–123.0)	87.1 (76.5–127.5)	110.0 (88.8–131.3)
**Triglycerides (mg/dL)**	65.5 (53.8–104.5)	121.5 (90.0–166.3)*	153.0 (120.0–198.5)*^#^
**AST (U/L)**	21.0 (16.5–23.0)	19.0 (17.0–21.0)	39.0 (26.0–56.0)*^#^
**ALT (U/L)**	17.0 (13.0–23.5)	18.0 (16.0–23.5)	41.0 (29.0–65.0)*^#^
**GGT (U/L)**	13.0 (10.0–20.0)	16.5 (10.3–22.8)	30.5 (16.0–54.1)*
**ALP (U/L)**	59.0 (48.0–69.0)	60.0 (51.0–74.0)	74.0 (60.3–81.5)*^#^
**HbA1c (%)**	5.0 (4.6–5.3)	5.2 (4.8–5.7)	5.8 (5.0–6.6)*^#^
**Iron (ug/dL)**	79.0 (59.8–104.8)	62.0 (45.3–71.5)	61.0 (36.0–78.5)*
**Transferrin (mg/dL)**	262.0 (226.0–287.0)	251.0 (231.3–264.8)	244.0 (220.5–264.0)*
**Transferrin saturation (%)**	18.3 (15.2–26.5)	13.8 (10.8–23.7)	23.1 (12.1–29.6)
**Ferritin (ng/mL)**	30.0 (19.5–74.2)	49.0 (27.0–73.0)	67.8 (33.5–175.0)*^#^
**Hepcidin (ng/mL)**	17.5 (5.8–26.7)	26.6 (14.5–44.3)*	23.5 (8.8–40.8)*
**CRP (mg/dl)**	<0.00001	1.5 (1.0–5.19) *	1.0 (1.0–2.0)*

ALT, alanine aminotransferase; ALP, alkaline phosphatase; AST, aspartate aminotransferase; BMI, body mass index; GGT, gamma-glutamyltransferase; HbA1c, glycosylated haemoglobin; HDL-C, high-density lipoprotein cholesterol; HOMA2-IR, homeostatic model assessment 2-insulin resistance; LDL-C, low-density lipoprotein cholesterol; NAFLD, non-alcoholic fatty liver disease; NL, normal liver; WC, waist circumference. Data are presented as the median (25th percentile–75th percentile).

* indicates significant differences with respect to normal-weight (p < 0.05);

^#^ indicates significant differences with respect to morbidly obese group with NL histology (p < 0.05).

### Evaluation of hepcidin circulating levels according to BMI and the presence of NAFLD

**Fig 1:**
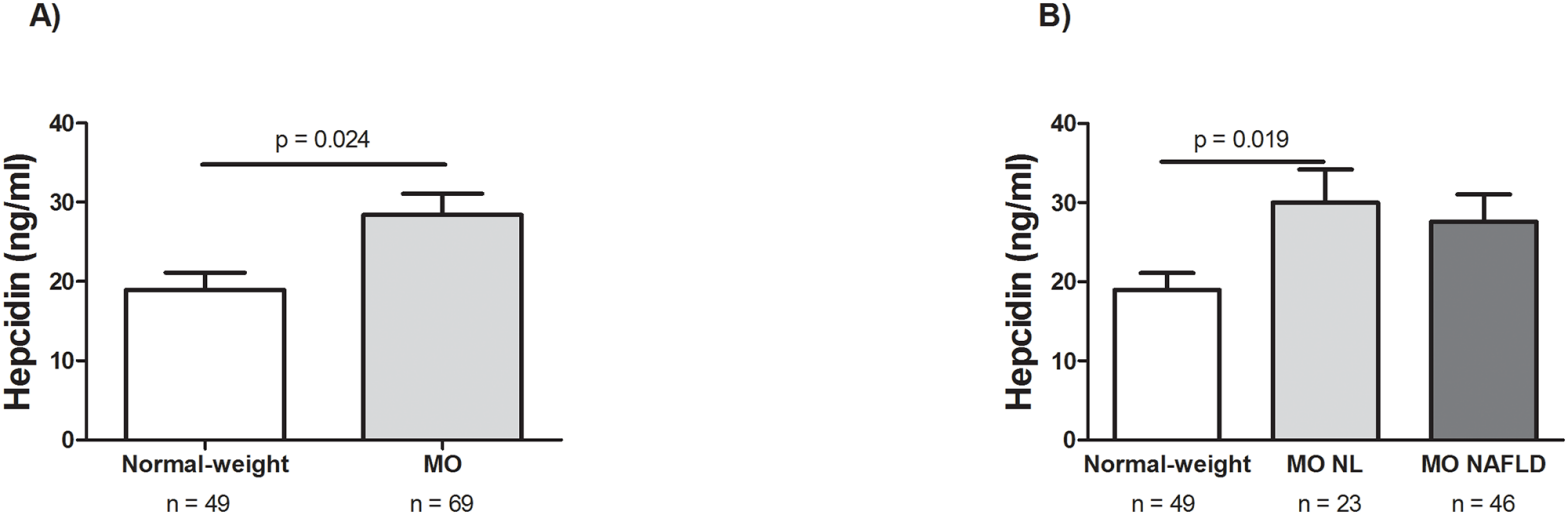
Hepcidin circulating levels. **(A)** Differential circulating hepcidin concentrations between normal-weight and morbidly obese women. **(B)** Hepcidin circulating levels in normal-weight women and in MO women according to liver histology into normal liver and NAFLD. MO, morbidly obese; NAFLD, non-alcoholic fatty liver disease. NL; normal liver. *p* < 0.05 were considered statistically significant.

The first objective of the present study was to evaluate the relationship between plasma hepcidin levels and the presence of NAFLD. To do this, we first compared the hepcidin circulating levels in our cohort of MO and normal-weight control women. The results indicate that the plasma hepcidin levels were significantly greater in morbidly obese than in normal-weight control women (Fig 1A). Then, we evaluated the hepcidin levels in our cohort of MO women according to the presence of NAFLD. We observed that the plasma levels of hepcidin were not significantly different in MO women with normal liver histology and those with NAFLD, but they were significantly greater in MO women with NL histology than in normal-weight subjects (Fig 1B). Finally, we also analyzed the hepcidin circulating levels in the MO NAFLD subgroup, but there were no significant differences between those with simple steatosis (SS) and NASH (MO SS: 22.16 (10.87–39.42); MO NASH: 25.00 (6.35–42.07); p = 0.811).

### Evaluation of the hepatic expression of genes involved in iron metabolism

**Fig 2:**
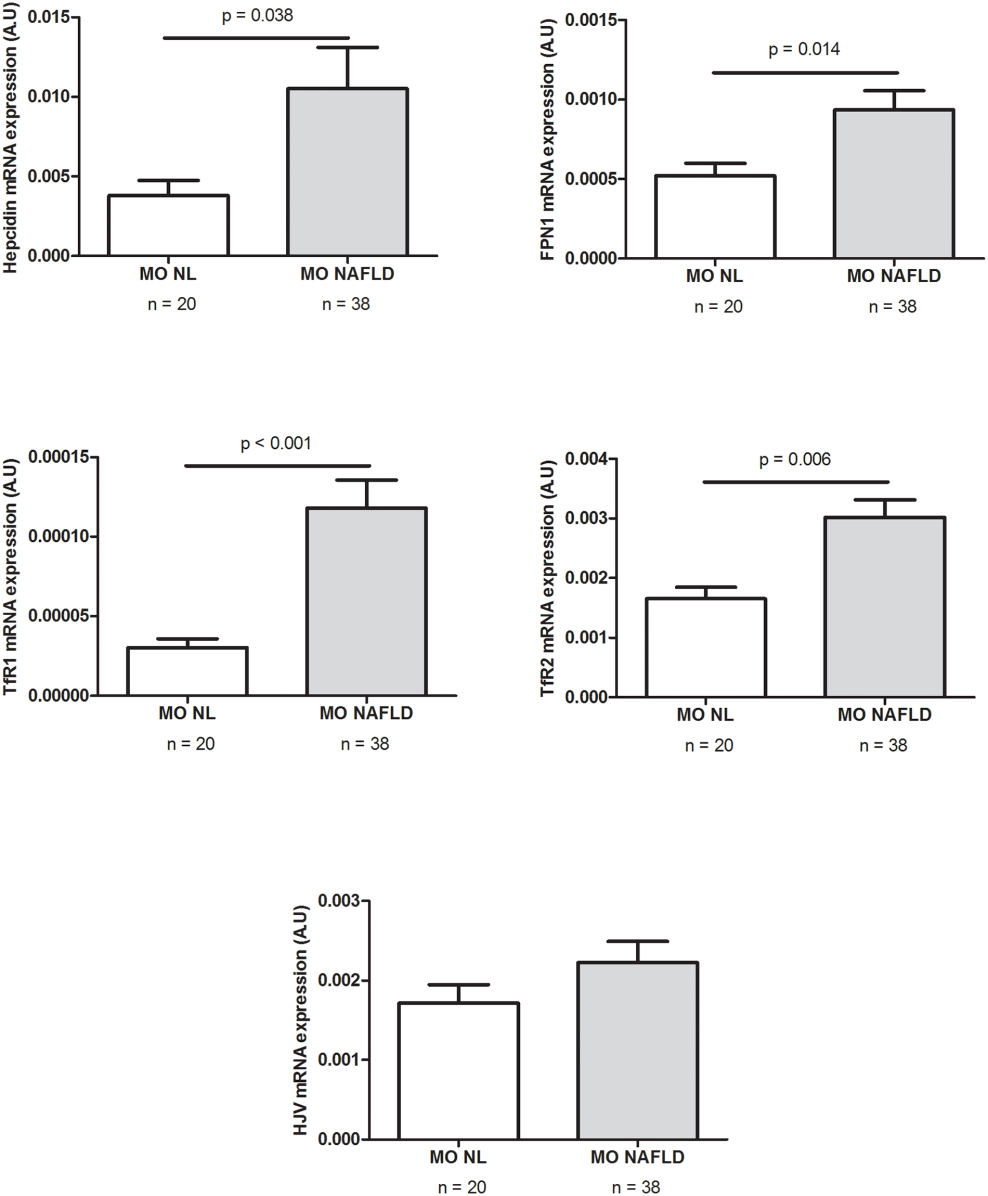
Differential hepatic expression of hepcidin and iron-related genes between MO women with normal liver histology and MO women with NAFLD. A.U; arbitrary units; MO, morbidly obese; NAFLD, non-alcoholic fatty liver disease. NL; normal liver. *p* < 0.05 was considered statistically significant.

**Fig 3:**
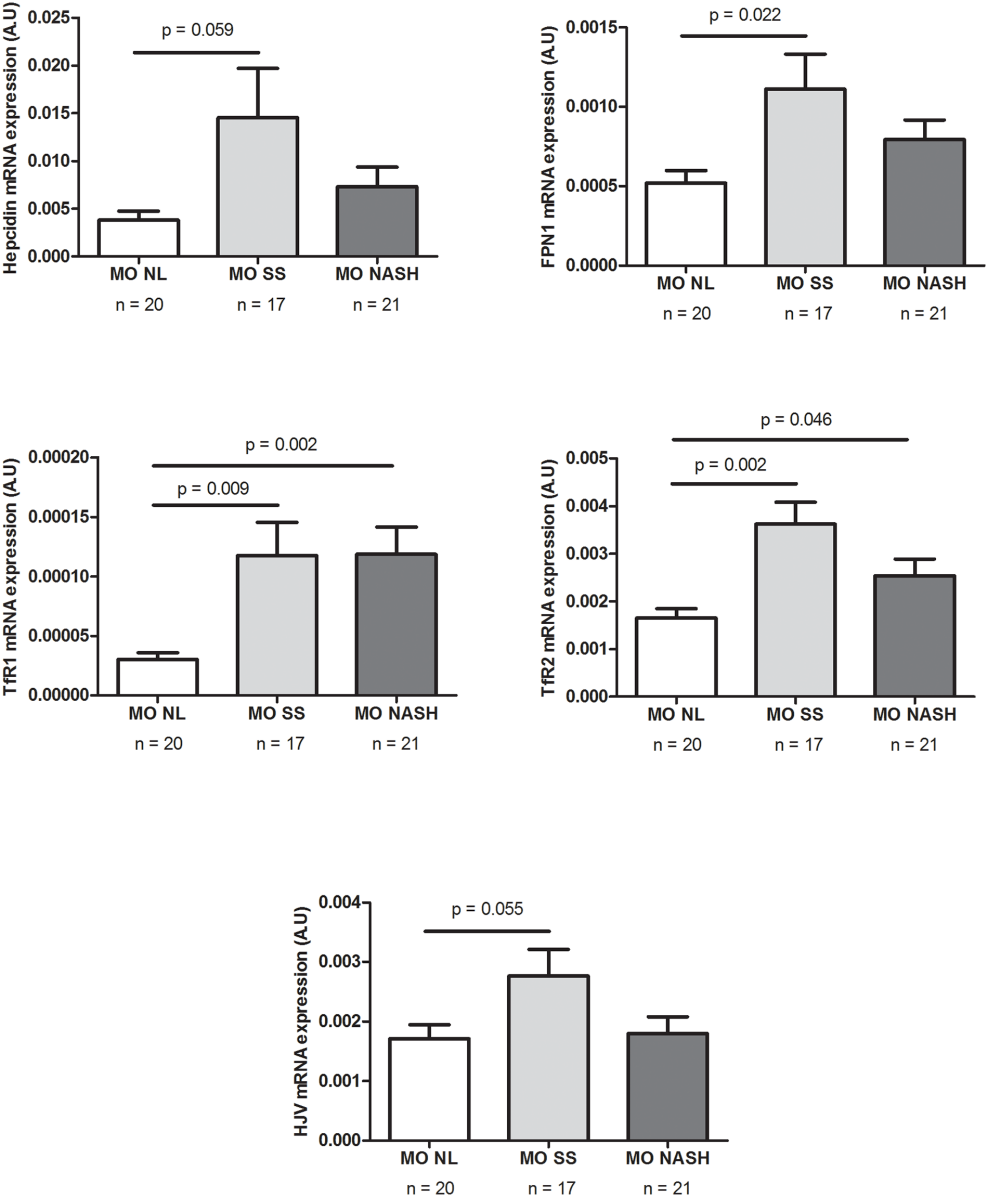
Hepatic expression in MO women according to liver pathology into NL, SS and NASH. A.U; arbitrary units; MO, morbidly obese; NAFLD, non-alcoholic fatty liver disease.NL; normal liver; NASH, non-alcoholic steatohepatitis; SS; steatosis. p < 0.05 is considered statistically significant.

The second objective of the present study was to evaluate the hepatic expression of hepcidin in our cohort of MO women and to analyze other genes involved in the iron metabolism (FPN1, TfR1, TfR2 and HJV). Although a lot of genes are involved in these pathways, we chose to measure FPN1, TfR1, TfR2 and HJV mRNA expression because mutations in Tfr2, HJV, and FPN1 prevent appropriate hepcidin response to iron, allowing increased absorption of dietary iron, and eventually iron overload [36]. We observed that in MO women with NAFLD the hepatic mRNA expression of hepcidin, FPN1, TfR1 and TfR2 was significantly greater than in MO women with normal hepatic histology (Fig 2). We categorized the MO NAFLD subjects in terms of their histological severity into simple hepatic steatosis and NASH. In this case, we did not observe any significant differences in the hepatic mRNA expression of the iron metabolism genes studied. However, the hepatic mRNA expression of FPN1, HJV, TfR1 and TfR2 was significantly higher in MO women with SS than in those with normal liver histology. Moreover, the hepatic mRNA expression of TfR1 and TfR2 was also significantly greater in NASH than in NL morbidly obese women (Fig 3).

### Correlations of hepcidin circulating levels with biochemical parameters

When we analyzed the association of hepcidin circulating levels with the biochemical parameters studied, we observed that plasma hepcidin correlated positively with ferritin (r = 0.708; p < 0.001), transferrin saturation (r = 0.230; p = 0.028) and iron (r = 0.274; p = 0.007) levels, but negatively with transferrin levels (r = -0.306; p = 0.003). In addition, we observed a negative association between hepcidin circulating levels and HDL-C (r = -0.198; p = 0.038), while a positive association with ALP was shown (r = 0.252; p = 0.009).

### Correlations of hepatic hepcidin and iron metabolism-related gene expression with genes involved in lipid metabolism, and biochemical parameters involved in iron metabolism

The results shown in Table 2 indicate that in our cohort of MO women the hepatic mRNA expression of hepcidin and the iron metabolism-related genes studied were positively associated with some key genes involved in the lipid metabolism. We also observed that hepcidin expression in liver correlated positively with FPN1 (r = 0.529; p < 0.001), TfR2 (r = 0.551; p < 0.001) and HJV (r = 0.593; p < 0.001). Moreover, hepatic hepcidin expression was not only positively associated with its circulating levels (r = 0.710; p < 0.001), but also with the plasma levels of iron, ferritin and transferrin saturation (Iron: r = 0.389; p = 0.014; Ferritin: r = 0.525; p < 0.001; Transferrin saturation: r = 0.471; p = 0.005). Finally, a strong negative association was observed between the hepatic expression of hepcidin and transferrin circulating levels (r = - 0.633; p < 0.001).

**Table 2:** Correlations of hepatic hepcidin and iron metabolism related genes expression with genes involved in lipid metabolism.

Variables	Hepcidin		FPN1		HJV		TfR1		TfR2	
	*r*	*p-Value	*r*	*p-Value	*r*	*p-Value	*r*	*p-Value	*r*	*p-Value
***Lipid metabolism genes***										
**LxRα**	**0.392**	**0.022**	**0.521**	**0.005**	**0.439**	**0.011**	**0.447**	**0.011**	**0.434**	**0.011**
**SREBP1c**	**0.380**	**0.022**	**0.384**	**0.019**	**0.389**	**0.022**	0.175	0.293	**0.377**	**0.022**
**ACC1**	0.021	0.888	0.116	0.513	-0.069	0.781	0.148	0.354	0.034	0.906
**FAS**	- 0.151	0.381	0.108	0.513	-0.102	0.667	0.242	0.164	-0.003	0.982
**PPARα**	**0.414**	**0.022**	**0.583**	**0.005**	**0.559**	**0.011**	**0.374**	**0.040**	**0.622**	**0.005**
**CPT1α**	0.186	0.332	0.276	0.094	0.318	0.057	0.231	0.164	**0.461**	**0.005**
**CROT**	0.156	0.381	0.273	0.094	0.243	0.169	0.317	0.082	0.318	0.057
**SREBP2**	0.138	0.392	0.173	0.330	0.044	0.839	0.247	0.164	0.261	0.121
**ABCA1**	0.224	0.240	0.026	0.863	0.010	0.946	0.022	0.881	0.074	0.763
**ABCG1**	0.245	0.213	**0.362**	**0.026**	0.123	0.628	**0.433**	**0.011**	0.255	0.121
**PNPLA3**	**0.431**	**0.022**	**0.418**	**0.019**	**0.397**	**0.027**	0.255	0.164	**0.434**	**0.016**

ABCA1, ATP-binding cassette transporter A1; ABCG1, ATP-binding cassette transporter G1; ACC1, acetyl-coenzyme A carboxylase 1; CPT1α, carnitine palmitoiltransferasa I; CROT, carnitine O-octanoyltransferase FAS, fatty acid synthase; LXRα, liver X receptor; PNPLA3, patatin-like phospholipase domain-containing protein 3; PPARα, peroxisome-proliferator-activated receptor α; SREBP1c, sterol regulatory element binding protein 1c; SREBP2, sterol regulatory element binding protein 2. p<0.05 is considered statistically significant.

*p-Value adjusted by Benjamini & Hochberg method -.

## Discussion

In our previous studies, we showed that lipogenesis seems to be downregulated in advanced stages of simple steatosis [4], as well as the hepatic lipid metabolism seems to be “controlled” by some miRNAs in patients with NAFLD [37]. As both the lipid and the iron metabolism seem to be involved in the pathogenesis of NAFLD [28,32], hepcidin seems to have a role in transition from steatosis to NASH [23] and lipid metabolism might be involved in hepcidin synthesis [28,32], in the present work we aimed to go one step further by exploring the relationship between plasma hepcidin levels, and the presence of NAFLD in morbidly obese patients, and also if any association between the hepatic expression of the main iron and lipid metabolism-related genes exists.

Although hepcidin has long been known to be linked with insulin-resistant states, including type 2 diabetes mellitus, the Metabolic Syndrome (MetS) and obesity [11,38], those studies that have examined the relationship between circulating hepcidin levels and the presence or severity of NAFLD have reported conflicting data. In this regard, in our study the plasma hepcidin levels were significantly greater in morbidly obese than in normal-weight control women. However, the plasma levels of hepcidin were not significantly different between MO women with normal liver histology and those with NAFLD. We confirm that obesity but not the presence of NAFLD was associated with circulating hepcidin levels, in agreement with other authors [31,39,40].

In a meta-analysis of obesity, Cheng *et al*. reviewed the association between adult obesity and iron homeostasis and found that obese subjects showed increased blood hemoglobin and serum ferritin concentration [41]. It is known that systemic iron homeostasis is controlled by hepcidin. In this regard, several studies have demonstrated that circulating hepcidin and ferritin concentrations are increased in obese subjects with low-grade chronic inflammation [11,42–49]. In our study, both hepcidin and CRP levels in MO women, regardless of their liver histology, were higher than those in normal-weight subjects.

With regard to NAFLD, although some authors have described higher serum levels of hepcidin in patients with NAFLD [25,27,30,39], others, according to our results, did not find any relationship between circulating levels of hepcidin and the presence of NAFLD [26,31,40]. These discrepancies could be explained by the different characteristics of the cohorts studied and the methods used. Our study was conducted in a cohort of morbidly obese women whereas Senates *et al*. [25] included a cohort of mildly obese patients of both genders. It should also be noted that Senates *et al*. do not provide the ferritin levels of the control group. As these levels have an influence on the levels of hepcidin, a bias could exist. Likewise, the study by Demircioglu *et al*., focused on overweight/mildly obese children and NAFLD was diagnosed by ultrasonography [30]. Finally, Zimmermann *et al*. included mildly obese patients of both genders with NASH and observed that hepcidin correlated with hepatic inflammation [27]. On contrary, we could not demonstrate any association between hepcidin circulating levels and the histological severity of NAFLD.

It is well known that obesity and NAFLD are associated with dysregulated lipid metabolism [3]. In fact, we have demonstrated elsewhere that there is a relation between a downregulation of the lipogenic pathway and the severity of steatosis in a cohort of MO women [4], and also between the liver expression of PNPLA3 and the severity of steatosis [50]. In addition, Barisani *et al*. and H. Mitsuyoshi *et al*. have reported that lipid metabolism might be involved in hepcidin synthesis [28,32]. Taking these results into account, the second goal of the present study was to evaluate the hepatic expression of hepcidin and other genes involved in iron metabolism, and to investigate if any association with lipid-metabolism related genes exists.

We observed that in our population of MO women, hepatic hepcidin expression was significantly higher in patients with NAFLD than in those with normal liver histology, and the same pattern was observed for the expression of important up-stream regulators of hepcidin expression, including transferrin receptor 1 (TfR1) and 2 (TfR2) and ferroportin 1 (FPN1). This finding is consistent with previous reports in which the hepatic levels of these genes in NAFLD patients were significantly higher than in controls, suggesting that the activity of FPN1 does not coincide with the amount of FPN1 [32,51]. Another recent study has reported increased hepatic hepcidin expression [23] in patients with NASH, suggesting that hepcidin plays a regulatory role in SS to NASH transition. Nevertheless, we did not observe any significant differences between NAFLD patients with simple steatosis and those with NASH. Regarding these discrepancies, it is important to note that these authors did not include a MO cohort, the BMI of the subjects studied was higher than 30 kg/m^2^ and lower than 40 kg/m^2^, and men were also included in the study.

Then, we also studied the relationship between some lipid-metabolism related genes and the expression of hepcidin and its up-stream regulators. The novelty of the study lies in the fact that our results show positive associations between hepcidin, hemojuvelin, FPN1, TfR1 and TfR2 hepatic expression with lipid metabolism-related genes involved in the *de novo* synthesis of fatty acids (LXRα and SREBP1c), fatty acid oxidation (PPARα), the secretion of lipoproteins (ABCG1), and the triacylglycerol lipase PNPLA3 in liver. In this regard, some authors have described the post-transcriptional regulation of human hepcidin genes by fatty acids in HepG2 cells [52]. Moreover, l different experimental models suggested that iron overload seems to have direct effects on hepatic lipid metabolism [53,54]. However, further human studies are necessary to clarify our findings because the association between the hepatic expression of the main iron and lipid metabolism-related genes can be casual. Perhaps, rather than a direct relationship, could exist a common pathogenic mechanism that causes the progression to NASH, as oxidative stress or inflammation [23].

We should point out that a limitation of this work is the lack of evaluation of protein expression. Additionally, the study is cross-sectional. We could not prove a causal link between iron and lipid metabolism-related genes in NAFLD. However, our study cohort of morbidly obese women has revealed a relationship between iron and lipid metabolism-related genes in NAFLD, without the interference of gender or age. Thus, our findings cannot be extrapolated to men or other obesity groups.

In summary, our results suggest that hepcidin circulating levels are associated with obesity but not with the presence of NAFLD. On the other hand, the hepatic expression of hepcidin and the expression of its important up-stream regulators could be related to lipid metabolism pathways in liver or at least sharing a common pathogenic mechanism, which might have implications for NAFLD pathogenesis. Further studies need to be performed in order to confirm or clarify these findings.
